# Oxygen Availability Influences Expression of *Dickeya solani* Genes Associated With Virulence in Potato (*Solanum tuberosum* L.) and Chicory (*Cichorium intybus* L.)

**DOI:** 10.3389/fpls.2018.00374

**Published:** 2018-03-21

**Authors:** Wioletta Lisicka, Jakub Fikowicz-Krosko, Sylwia Jafra, Magdalena Narajczyk, Paulina Czaplewska, Robert Czajkowski

**Affiliations:** ^1^Department of Biotechnology, Intercollegiate Faculty of Biotechnology of University of Gdañsk and Medical University of Gdañsk, University of Gdañsk, Gdañsk, Poland; ^2^Laboratory of Electron Microscopy, Faculty of Biology, University of Gdañsk, Gdañsk, Poland; ^3^Laboratory of Mass Spectrometry, Core Facility Laboratories, Intercollegiate Faculty of Biotechnology of University of Gdañsk and Medical University of Gdañsk, University of Gdañsk, Gdañsk, Poland

**Keywords:** hypoxia, Tn5 transposon mutagenesis, virulence, abiotic stress, colonization, anaerobic conditions

## Abstract

*Dickeya solani* is a Gram-negative necrotrophic, plant pathogenic bacterium able to cause symptoms in a variety of plant species worldwide. As a facultative anaerobe, *D. solani* is able to infect hosts under a broad range of oxygen concentrations found in plant environments. However, little is known about oxygen-dependent gene expression in *Dickeya* spp. that might contribute to its success as a pathogen. Using a Tn5 transposon, harboring a promoterless *gusA* reporter gene, 146 mutants of *D. solani* IPO2222 were identified that exhibited oxygen-regulated expression of the gene into which the insertion had occurred. Of these mutants 114 exhibited higher expression under normal oxygen conditions than hypoxic conditions while 32 were more highly expressed under hypoxic conditions. The plant host colonization potential and pathogenicity as well as phenotypes likely to contribute to the ecological fitness of *D. solani*, including growth rate, carbon and nitrogen source utilization, production of pectinolytic enzymes, proteases, cellulases and siderophores, swimming and swarming motility and the ability to form biofilm were assessed for 37 strains exhibiting the greatest oxygen-dependent change in gene expression. Eight mutants expressed decreased ability to cause disease symptoms when inoculated into potato tubers or chicory leaves and three of these also exhibited delayed colonization of potato plants and exhibited tissue specific differences in gene expression in these various host tissues. The genes interrupted in these eight mutants encoded proteins involved in fundamental bacterial metabolism, virulence, bacteriocin and proline transport, while three encoded hypothetical or unknown proteins. The implications of environmental oxygen concentration on the ability of *D. solani* to cause disease symptoms in potato are discussed.

## Introduction

Soft Rot *Enterobacteriaceae* (SRE: *Pectobacterium* spp. and *Dickeya* spp.) are important phytopathogenic, pectinolytic bacteria that cause large and increasing economic losses in agricultural crops worldwide (Pérombelon, 2002; Kado et al., 2006). In potato (*Solanum tuberosum* L.), *Pectobacterium* spp. and *Dickeya* spp. are responsible for tuber soft rot in transit and storage as well as potato blackleg in plants in the field (Pérombelon, 2002; Charkowski, 2006). The major source of inoculum for soft rot and blackleg bacteria are (latently) contaminated potato tubers that transfer cells between different locations as well as from one growing season to the next (Pérombelon, 1992; Toth and Birch, 2005). While tuber contamination with SRE can occur during plant growth in the field, harvesting and grading are considered the most important stages at which healthy potato tubers acquire bacteria. This process most commonly occurs when rotten tubers harboring high densities of inoculum are present (Van Der Wolf and De Boer, 2007).

Even if a majority of tubers may become contaminated internally via vascular tissue- inoculum or externally via contamination of periderm with *Pectobacterium* spp. and *Dickeya* spp., extensive tuber rotting during storage is rare, as the bacteria remain dormant unless conditions prevail that allow their multiplication (Pérombelon and Lowe, 1975). Rotting in storage and transit is almost exclusively linked to poor ventilation and high humidity which together lead to the creation of a water film on tuber surface, which restricts the diffusion of oxygen, causing at least local oxygen depletion (hypoxia) (Kotoujansky, 1987).

It is believed that the oxygen status of the tuber is one of the critical environmental factors governing soft rot susceptibility and symptom progression upon contamination with SRE (De Boer and Kelman, 1978). Several reports documented that the occurrence and severity of potato soft rot caused by pectinolytic bacteria increased under oxygen-limited or anaerobic conditions (Perombelon and Kelman, 1980). In the past, this was almost always attributed to decline of host resistance, characterized as an alteration of oxygen-dependent wound healing resulting from inhibition of gene expression in the tubers and lack of antibacterial protein synthesis (Butler et al., 1990). It has been also suggested that oxygen-limited conditions may increase the relative fitness of the pathogen during infection as *Pectobacterium* spp. and *Dickeya* spp. are both able to grow under anaerobic conditions (Charkowski, 2006) and hence, may more efficiently compete with co-occurring aerobic (antagonistic) bacteria where oxygen availability is low. Furthermore, Perombelon and Kelman (1980) reported that a lack of oxygen can trigger a rapid expansion of bacterial cells in latently infected plant parts leading to massive post-harvest tuber loss due to soft rot (Perombelon and Kelman, 1980).

Despite the considerable study of the ecology and physiology of bacteria in low-oxygen environments, little is known about oxygen-dependent gene expression in pectinolytic *Dickeya* spp. Most study of this taxon has been restricted to *D. dadantii* reference strain 3937 (Hugouvieux-Cotte-Pattat et al., 1992; Babujee et al., 2012). Hugouvieux-Cotte-Pattat et al. (1992) reported that the production of pectate lyase, one of the key macerating enzymes of *Pectobacterium* spp. and *Dickeya* spp., is induced under anaerobic conditions. Furthermore, transcriptional profiling revealed that ca. 10% of *D. dadantii* 3937 genes are differentially expressed under oxygen-limited conditions in comparison with normal oxygen concentration (so-called normoxia) (Babujee et al., 2012). As far as we are aware, no data are available on oxygen-dependent gene expression in other SRE species such as *Dickeya solani* that differs substantially genetically from *D. dadantii*.

Since 2000, *D. solani* has been associated with large and increasing losses in seed and edible potato production due to both soft rot and blackleg in Europe (Toth et al., 2011; Degefu et al., 2013). This pathogen has been isolated from both potato plants and tubers in many European countries (Toth et al., 2011; Van Der Wolf et al., 2014) as well as in Israel (Tsror et al., 2010), Georgia (Tsror et al., 2011), Turkey (Ozturk and Aksoy, 2017), and Brazil (Cardoza et al., 2016). The rapid spread of *D. solani* throughout Europe is almost certainly due to the fact that this species is more virulent than the close-related *D. dianthicola* and *P. atrosepticum*, that were the most well-established pectinolytic potato pathogens in European potato ecosystem in the past (Janse and Ruissen, 1988; Toth et al., 2011; Czajkowski et al., 2012).

The purpose of this study was to identify and characterize the *D. solani* genes that are differentially expressed under hypoxia compared to normal oxygen conditions by a process of random mutagenesis of *D. solani* genome using Tn5 transposon with promoterless *gusA* reporter gene and to associate such genes with oxygen-dependent *D. solani* phenotypes including virulence to plants. The implications of such oxygen-dependent bacterial fitness genes are discussed.

## Materials and Methods

### Bacterial Strains and Culture Conditions

*Escherichia coli* strain S17 λ-pir carrying plasmid pFAJ1819 with mini-Tn5 transposon (Xi et al., 1999) was cultured in tryptic soy broth (TSB; Oxoid) supplemented with neomycin (Sigma-Aldrich) to a final concentration of 50 μg mL^-1^ in shaken cultures (200 rpm) or on tryptic soy agar (TSA; Oxoid) at 37°C for ca. 16 h. *Dickeya solani* strain IPO2222 (Van Der Wolf et al., 2014) was grown at 28°C in TSB or in M9 minimal medium (per liter: 6 g Na_2_HPO_4_, 3 g KH_2_PO_4_, 0.5 g NaCl, and 1 g NH_4_Cl) supplemented with glucose (Sigma-Aldrich) to a final concentration of 0.4%. To solidify the media, 15 g L^-1^ agar (Oxoid) was added. If required, the growth media were supplemented with neomycin to a final concentration of 50 μg mL^-1^ and with X-gluc (5-bromo-4-chloro-3-indolyl-b-D-glucuronide; GeneON) to a final concentration of 20 μg mL^-1^.

### Transposon Mutagenesis With Mini-Tn5 Transposon, Determination of the Transposon Transfer Rate and Estimation of the Genome Coverage With the Mutagenesis

Random transposon mutagenesis via conjugation of *D. solani* strain IPO2222 with *E. coli* strain S17 λ-pir pFAJ1819 was done as described previously (Czajkowski et al., 2011, 2017a). Plasmid pFAJ1819 contains a mini-Tn5 transposon with a promotorless *gusA* gene. This plasmid can be stably replicated in *E. coli* S17 λ-pir but not in *D. solani* cells (Wilson et al., 1995; Xi et al., 1999). The Tn5 transfer rate (conjugation rate of pFAJ1819 plasmid from *E. coli* to *D. solani* cells) was determined as previously described (Czajkowski et al., 2017a) using the equation: *X* = (*I*_r_ × 100)/*I*_d_; where *X* is the conjugation rate, *I*_r_ is recombinant cfu/mL of conjugation mixture and *I*_d_ is donor cfu/mL of conjugation mixture. The experiment was independently repeated three times and the results were averaged. To determine the coverage of the IPO2222 genome with the Tn5 transposition events in the mutagenesis assays, the Clark-Carbon equation [*P* = 1-(1-*f*)ˆ*N*] was used (Pérez-Ortín et al., 1997), where *P* – is probability to find a gene with desired function, *f* – fraction of the genome [assuming that an average gene in *D. solani* is 1200 bp.-long and that the *D. solani* genome is 4919833 bp. (Khayi et al., 2016) *f* = 1200/4919833 = 0.00024391] and *N* – the number of tested IPO2222 Tn5 mutants, in our experimental design, *N* = 10000.

### Identification of *D. solani* Tn5 Mutants by PCR and Plating on Selective CVP Medium

PCR detection of *D. solani* Tn5 mutants was performed according to Laurila et al. (2010) using primers Df (5′- AGAGTCAAAAGCGTCTTG-3′) and Dr (5′-TTTCACCCACCGTCAGTC-3′). These primers amplify a 133 bp fragment exclusively from strains of *Dickeya* species (Laurila et al., 2010). Amplified DNA fragments were detected by electrophoresis on a 1% 0.5 × TBE agarose gel stained with 50 μg mL^-1^ GelRed (Biotium). The ability of Tn5 mutants to form cavities (pits) on crystal violet pectate medium (CVP) was tested as described by (Hélias et al., 2011).

### Glucuronidase Activity of *D. solani* Tn5 Mutants

Glucuronidase activity of the Tn5 bacterial mutants was visually inspected 48 h post-incubation by a change in blue color intensity of bacterial colonies growing on M9 agar plates supplemented with neomycin to a final concentration of 50 μg mL^-1^ and X-gluc to a final concertation of 20 μg mL^-1^ under normoxic conditions or under oxygen-limited conditions generated with the use of GasPak EZ Large Incubation Container with GasPak EZ Gas Generating Sachets (BD Diagnostics) using protocol provided by the manufacturer. The color intensity of the colonies growing under normoxic and hypoxic conditions was compared daily.

### Semi-quantitative Assays to Assess the Rate of Gene Expression in *D. solani* Tn5 Mutants by Measuring GUS Activity

Glucuronidase (GUS) activity was quantified by a spectrophotometric assay with p-nitrophenol-b-D-glucuronide (Sigma-Aldrich) as a substrate for glucuronidase as previously described (Czajkowski et al., 2017a). Total protein content was determined according to the Bradford method (Bradford, 1976) using a BCA protein assay kit (Pierce). Glucuronidase activity of the Tn5 mutants was measured as pmol product (p-nitrophenol)/min/μg total protein. Tn5 mutants showing a statistically significant increase of glucuronidase activity under normoxic vs. hypoxic or hypoxic vs. normoxic conditions (at least 1.5-fold) were retested under the same conditions with four replicates per isolate and treatment, and selected for further experiments.

### Southern Hybridization Analyses of the Tn5 Insertions

In order to determine the number of Tn5 insertions per genome, Southern hybridization analyses were performed as described earlier (Sambrook et al., 1989). Briefly, genomic DNA from selected *D. solani* Tn5 mutants was isolated using Wizard Genomic DNA Purification Kit (Promega) as described by the manufacturer. Southern blot transfer of PstI-digested bacterial genomic DNA was performed according to Sambrook et al. (1989). A 679 bp. PCR product of the *gusA* gene was used as the hybridization probe and the hybridization and detection were performed according to the protocol of the digoxigenin DNA-labeling and detection kit (Roche Diagnostics GmbH).

### Sequencing of the Genomes of Selected Tn5 *D. solani* Mutants, Identification of the Tn5 Insertion Site in Genome and Determination of the Function of Tn5-Distrupted Genes

In order to precisely localize the Tn5 insertion site in a genome, the bacterial genomic DNA obtained as described above was sequenced and assembled at the Laboratory of DNA Sequencing and Oligonucleotide Synthesis at the Institute of Biochemistry and Biophysics of the Polish Academy of Science, Warsaw, Poland using the Illumina technology. Structural and functional annotations were obtained from RAST (Rapid Annotation using Subsystem Technology, accessed via the internet http://rast.nmpdr.org/). The location of the Tn5 transposon insertions in the genomes of *D. solani* IPO2222 mutants was determined with the use of BLASTN and BLASTX alignments accessed via http://blast.ncbi.nlm.nih.gov/Blast.cgi as previously described (Czajkowski et al., 2017a). Per mutant, at least 1000 bp flanking regions of the Tn5 insertion were analyzed in order to evaluate the genomic context of the Tn5-distruped gene. Likewise, the putative molecular function of *D. solani* Tn5 disrupted genes was determined with the use of BLASTN and BLASTX alignments accessed via http://blast.ncbi.nlm.nih.gov/Blast.cgi. Additionally, the function of unknown genes (hypothetical genes and open reading frames coding for unknown proteins) was predicted using GeneSilico Protein Structure Prediction meta-server^1^, containing known three-dimensional (3D) protein structures (Kurowski and Bujnicki, 2003), together with PSI-BLAST accessed from the NCBI website^2^. The predicted functions with the highest scores were considered as the most valid.

### Evaluation of Colony Morphology of *D. solani* Tn5 Transposon Mutants

Selected *D. solani* IPO2222 Tn5 mutants were tested for putative changes in a colony morphology resulted from a disruption of a gene function by the Tn5 presence in the bacterial genome. For this, overnight bacterial cultures (ca. 10^9^ cfu mL^-1^) grown in TSB at 28°C with shaking (200 rpm) were washed twice with 1/4 Ringer’s buffer. Bacterial suspensions were adjusted to OD_600_ 0.1 (ca. 10^8^ cfu mL^-1^). 3 μL of such prepared bacterial cultures in duplicates were placed on the surface of M9 agar plates supplemented with glucose to a final concentration of 0.4%. Inoculated plates were incubated for 48h at 28°C and the resulting bacterial colonies were analyzed with the use of Leica MZ10F stereomicroscope at 10 × and 40 × magnification and Leica DFC450C camera system for the colony morphology and the diameter. *D. solani* strain IPO2222 was used as a control. The experiment was independently repeated once with the same setup. At least ten photographs were taken per mutant and wild type strain and per experiment to estimate the bacterial colony diameter.

### Assessment of Tn5 *D. solani* Mutant Morphology Under Transmission Electron Microscope (TEM)

To assess the morphology of *D. solani* Tn5 mutants, bacteria were grown overnight in TSB at 28°C with shaking (200 rpm). For the TEM analysis, bacteria were adsorbed onto carbon-coated grids (Sigma-Aldrich) stained with 1.5% uranyl acetate and directly examined with an electron microscope (Tecnai Spirit BioTWIN, FEI) as described previously (Czajkowski et al., 2015, 2017b). At least ten photos were taken per analyzed mutant and wild type strain.

### Determination of Growth Rate of *D. solani* Tn5 Mutants *in Vitro*

To assess bacterial growth, Tn5 overnight bacterial cultures with a density of ca. 10^9^ cells mL^-1^ in M9 medium supplemented with 0.4% glucose and 50 μg mL^-1^ neomycin were diluted 50 times in the same fresh medium but without antibiotic supplementation. Five hundred microliters of diluted bacterial cultures were aseptically transferred to the sterile wells of 48-well microtitre plates (BD Labware) and these were sealed with optically transparent sealing tape (Sarstedt) to prevent from contamination and evaporation of bacterial cultures. Growth rate was determined at 28°C by measuring the optical density (OD_600_) automatically every hour in an EnVision Multilabel Reader (Perkin Elmer) for a total period of 16 h. Bacterial cultures in 10 mm wells were shaken in an orbital shaker at 60 rpm, with a shake duration of 1 h between OD measurements to prevent anaerobic conditions from occurring and a creation of bacterial sedimentation. The growth of each Tn5 *D. solani* mutant was analyzed in six replicates and the results were averaged. Each 48-well plate used contained six negative (sterile growth medium) and six positive (wild type *D. solani* IPO2222 culture) wells as controls. The experiment was independently repeated once with the same setup.

### Phenotypic Characterization of *D. solani* Tn5 Mutants Using Plate Assays

*Dickeya solani* Tn5 mutants showing at least 1.5-fold (equivalent to 150% increase) change in the relative gene expression at normoxic conditions to gene expression at oxygen-limited (hypoxic) conditions (and *vice versa*) were screened for various phenotypic features including swimming and swarming motility, the ability to grow on the TSA medium supplemented with 5% NaCl (Dickey, 1979), cellulases (Py et al., 1991), proteases (Ji et al., 1987), pectinolytic enzymes (Perombelon and Van Der Wolf, 2002) and siderophores (Schwyn and Neilands, 1987) and for ability to form biofilm (Nykyri et al., 2013). As well, the ability to cause rotting of chicory leaves and potato tubers was evaluated as described in (Czajkowski et al., 2012; Krzyzanowska et al., 2012).

### Phenotypic Characterization of *D. solani* Tn5 Mutants Using BIOLOG Phenotypic Microarrays GEN III

Selected *D. solani* IPO2222 Tn5 transposon mutants were tested using a BIOLOG phenotypic microarray system with GEN III microplates (Biolog Inc.). Each BIOLOG GEN III plate contains 94 phenotypic tests: 71 carbon source utilization assays and 23 chemical sensitivity assays as described by the manufacturer. To test *D. solani* mutants, fresh bacterial cultures were grown on TSA for 24 h at 28°C and were used to inoculate into inoculation fluid (IF-A) by using sterile cotton swab. Turbidity of the inoculants was adjusted to ca. 90% T of IFA with the use of turbidimeter as suggested by the manufacturer. The suspensions of 100 μl were inoculated into each well of the GEN III microplates using multichannel pipette. Inoculated plates were sealed with parafilm and incubated for 24 h at 28°C and after this time the wells were observed for color development (positive reaction) by eye. Color development was also recorded using Wallac Victor 2 Micro-plate Reader (Perkin Elmer) using a 405 nm wavelength filter. Per *D. solani* mutant, two BIOLOG GEN III plates were used. *D. solani* strain IPO2222 was used as a control.

### Phenotypic Characterization of *D. solani* Tn5 Mutants Using API-ZYM Tests

To test if Tn5 insertion affects the profile of extracellular *D. solani* enzymes (Humble et al., 1977), selected *D. solani* Tn5 mutants were tested with the use of API-ZYM stripes (bioMérieux) following the protocol provided by the manufacturer. The experiment was repeated one time with the same setup.

### Phenotypic Characterization of *D. solani* Tn5 Mutants Using Whole Cell MALDI-TOF MS Analysis

Selected *D. solani* IPO2222 Tn5 transposon mutants were tested using a whole-cell MALDI-TOF MS spectral analysis as previously described (Vandroemme et al., 2013). Briefly, IPO2222 wild type and selected Tn5 mutants were grown on M9 medium supplemented with glucose to a final concentration of 0.4% at 28°C for 24 h prior to analysis. As a matrix ferulic acid (FA) (10 mg/ml) in 17% formic acid, 33% acetonitrile, 50% water was used. In each case, a 0,6 μl of matrix solution was used to overlay the sample spot, and the plate was then left to crystallize at room temperature. Directly after spot preparation (ca. 15 min), protein mass fingerprints were obtained using an 5800 MALDI-TOF/TOF mass spectrometer (AB Sciex, Framingham, MA, United States), with detection in the linear middle mass (4000–20 000 Da), positive ion mode for a total of 1000 laser shots by an 1 kHz OptiBeam laser (YAG, 349 nm). Laser intensity remained fixed for all screened samples. Registered spectra were analyzed with Data Explorer software (AB Sciex). All MALDI-TOF MS spectra used in this study were averages of at least four replicated measurements per analyzed strain.

### Screening for Plant Tissue-Induced Gene Expression of *D. solani* Tn5 Mutants

Selected *D. solani* Tn5 transposon mutants in genes differentially regulated under hypoxic/normoxic and normoxic/hypoxic conditions were screened for plant-tissue induced gene expression using a previously described protocol (Czajkowski et al., 2017a; Fikowicz-Krosko and Czajkowski, 2017). For each of the Tn5 *D. solani* mutant to be screened, in duplicates, leaf, stem, root cuts and minitubers were analyzed. For control, per mutant, wells containing growth medium and inoculated with respective Tn5 mutant but without plant tissue were used. A Tn5 *D. solani* WN2 mutant with constitutive glucuronidase expression (Fikowicz-Krosko and Czajkowski, 2017) was used as a positive control for glucuronidase activity as previously described (Goyer and Ullrich, 2006). The experiment was repeated independently one time with the same setup.

### Host Colonization and Virulence of *D. solani* Tn5 Mutants on Potato Plants Grown in Tissue Cultures

*In vitro* potato plants cv. Kondor were cultivated and propagated on Murashige and Skoog (MS) medium with 30 g L^-1^ sucrose and 7 g L^-1^ agar in culture tubes and under temperature and light regime as previously described (Czajkowski et al., 2015; Murashige and Skoog, 1962). As a negative control, 10 μl of sterile demineralized water was used. Each treatment consisted of 10 potato plants grown in individual culture tubes and the entire experiment was repeated independently one time with the same setup (*n* = 20 per treatment). Inoculated plants were visually inspected after 6 and 16 days post inoculation (dpi) for wilting, typical blackleg symptoms, stem desiccation and/or plant death as described previously (Czajkowski et al., 2015).

### Statistical Analyses

Bacterial count data were analyzed by ordinary linear regression using the statistical software package GenStat (Payne et al., 2007). To achieve the approximate normality, data were log transformed after adding value 1 to avoid taking logs of zero. Results were considered to be significant at *p* = 0.05 and pair-wise differences were obtained using the *t*-test. For experiments involving *in vitro* plants, data were analyzed according to the experimental design in which two replicated experiments were done per each time point with treatments of replicated plants. The adopted linear model was a complete block design with replicates as complete blocks, main effects analyzed for time and treatment and the two-way interaction between time and treatment. Normal distribution was assumed for plant height and weight.

## Results

### Transposon Mutagenesis, Frequency of Tn5 Transposon Transfer and Quantification of Glucuronidase Activity

A total of 10,000 Tn5 transposon mutants of *D. solani* strain IPO2222 were screened for differential expression of the gene into which the transposon had inserted in cells exposed to the presence and absence of oxygen. The estimated frequency of Tn5 transposon transfer from the donor *E. coli* λ-pir to recipient *D. solani* IPO2222 was ca. 10^-6^ cells/recipient. Glucuronidase (GUS) activity was assessed visually on a solid M9 minimal medium. Of the 10,000 mutants tested, only 146 (1.46%) exhibited differences in their GUS activity when compared under normal oxygen conditions to anaerobic conditions. From the 146 Tn5 mutants expressing differential GUS activity, 114 (78%) exhibited a higher expression under normal oxygen conditions while only 32 (22%) exhibited a higher apparent expression under oxygen-limited conditions (**Table 1**). Given the number mutants assessed and the genome size of *D. solani* we estimate using the Clark-Carbon equation that approximately 90% of the genes in *D. solani* IPO2222 harbored at least one insertion of the reporter transposon. The degree of oxygen-dependent differential expression of the GUS reporter gene differed among the 146 mutants as determined by spectrophotometric GUS analysis. While the absolute level of gene expression as measured by GUS analysis differed widely (1–68 U mg^-1^ total protein), 37 of the mutants exhibited at least a 1.5-fold (150%) change in relative gene expression when grown in normal oxygen compared to hypoxic conditions; 8 mutants exhibited higher expression under normal oxygen conditions while 29 mutants exhibited higher expression under anorexic conditions. These 37 mutants were thus chosen for more detailed phenotypic and genetic analysis.

**Table 1:** Workflow of the selection of oxygen-dependent *Dickeya solani* IPO2222 Tn5 mutants (details provided in the text).

No. total generated Tn5 *D. solani* mutants	No. mutants with differential gene expression due to oxygen availability	No. mutants with at least 1.5-fold (150%) difference in gene expression due to the oxygen availability	No. mutants expressing phenotypes *in planta*	Average frequency of Tn5 transposon transfer between transposon donor and recipient
10 000	146	37	8	10^-6^ cells/recipient

### Characterization of Phenotypic Features of *D. solani* Tn5 Transposon Mutants

The 37 Tn5 mutants exhibiting substantial differential oxygen dependent gene expression were screened in plate assays for phenotypes distinct from the phenotype of the wild type IPO2222 strain. No differences were observed between the tested Tn5 mutants and *D. solani* IPO2222 WT strain in their ability to cause cavities to form on CVP medium, production of pectinolytic enzymes, cellulases, proteases and siderophores, swimming and swarming motility and growth on 5% NaCl.

The mutants also did not differ in growth rate (Supplementary Figure 1) and protein mass fingerprints, with the exception of the mutant B18 that lacked a peak of 18472.5 (m/z) present in fingerprints of all other analyzed Tn5 mutants and wild type strain (Supplementary Figure 2). The peak of 18472.5 (m/z) present in IPO2222 wild type strain and absent in B18 mutant could not be, however, directly related to the estimated protein mass of the mutated WP_022634983 locus (22993 Da) (**Table 2**). This suggests that it is rather a different protein or protein fragment, not an intact full-length protein mutated by the presence of the Tn5 in B18 mutant.

**Table 2:** Phenotypes of genetic loci regulated by the oxygen availability of eight *D. solani* IPO2222 Tn5 mutants.

		Phenotypic features						Relative GUS expression		
No	Mutant	Colony morphology	Cell morphology	Virulence on potato tubers	Virulence on chicory leaves	Tn5 locus, accession number, protein, predicted function	Homologs in other *Dickeya* spp.	U/mg total protein	Inducing oxygen status	Fold induction
1	A49	wt^a^	wt	wt	decreased^b^	WP_022634006; LacI/PurR family transcriptional regulator	*D. zeae, D. dadantii, D. dianthicola, D. chrysanthemi*	2.0	hypoxia	2.0
2	A57	wt	wt	wt	decreased	WP_022634533; lytic transglycosylase, murein transglycosylase D	*D. dadantii, D. dianthicola, D. zeae, D. chrysanthemi*	9.0	normoxia	1.6
3	A65	wt	wt	wt	decreased	WP_022634250; ferric vibriobactin enterobactin ABC transport system ATP-binding protein ViuC/FecE	*D. zeae, D. dadantii, D. dianthicola, D. chrysanthemi, D. paradisiaca*	10.0	normoxia	3.6
4	A68	wt	no flagella	decreased	decreased	WP_022633687; hypothetical, inner membrane protein	*D. zeae, D. dadantii,D. dianthicola, D. chrysanthemi*,	67.0	hypoxia	2.0
5	A77	wt	wt	wt	decreased	WP_012883918; colicin transporter, TolR protein	*D. dianthicola, D. paradisiaca, D. chrysanthemi, D. zeae, D. dadantii*,	2.0	hypoxia	1.9
6	B7	small, creamish, white, crateriform with entire margin	wt	decreased	decreased	WP_023637659; proline-specific permease ProY	*D. dadantii, D. dianthicola, D. zeae, D. chrysanthemi, D. paradisiaca*	68.0	normoxia	1.8
7	B18	small, creamish, white, crateriform with entire margin	elongated cells	decreased	decreased	WP_022634983; hypothetical protein	*D. dadantii, D. zeae, D. chrysanthemi*	3.0	normoxia	2.1
8	B29	wt	wt	decreased	wt	WP_022634128; hypothetical protein	*D. dadantii, D. zeae*	4	normoxia	1.95

*^*a*^not significantly different than in *D. solani* strain IPO2222 wild type; ^*b*^statistically significantly lower than in *D. solani* strain IPO2222 wild type.*

**FIGURE 1:**
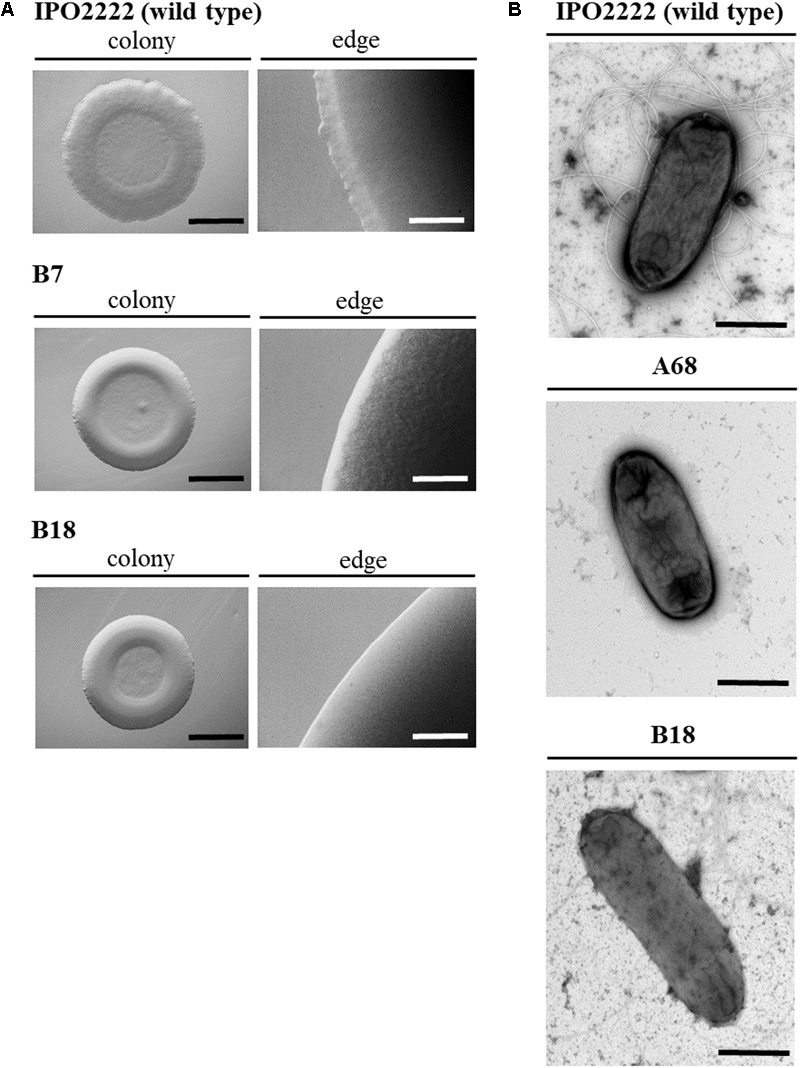
Morphology of *Dickeya solani* IPO2222 Tn5 transposon mutants B7 and B18 colonies grown on M9 agar with glucose for 24 h at 28°C visualized with stereomicroscopy with low magnification (10–40×); size bar: black – 150 mm, white – 5 mm **(A)** and individual bacterial cells visualized by transmission electron microscopy (TEM); size bar: 1000 nm. TEM analyses were conducted on wild type and Tn5 mutant cells of *D. solani* IPO2222 grown overnight in Tryptone Soya Broth (TSB) with shaking (200 rpm) at 28°C. Photos were taken directly after bacteria collection from liquid cultures. For this, bacteria were adsorbed onto carbon-coated grids (Sigma) stained with 1.5% uranyl acetate and directly examined with electron microscope (Tecnai Spirit BioTWIN, FEI) **(B)**. The figure shows representative colonies and cells.

Plating techniques combined with stereomicroscopy were used to assess if the presence of Tn5 insertion in *D. solani* genomes affected the morphology of 37 mutant colonies grown on M9 agar plates. The majority of mutant colonies exhibited a colony phenotype similar to the one of the wild type *D. solani* strain one examined by eye and under a stereoscopic microscope, being ca. 0.5 cm in diameter, circular with undulate margin, crateriform and opaque. Two mutants (B7 and B18) in repeatable experiments, however, formed smaller (ca. 0.3–0.4 cm in diameter), white cream-colored, opaque and crateriform, circular with entire margin colonies on M9 + glucose agar plates, distinct from the colonies of the WT strain (**Figure 1A**). In transmission electron microscopy (TEM) analysis, of 8 mutants tested, 6 showed no visible differences in bacterial cell morphology in comparison with the WT strain (data not shown). However, mutant B18 possessed elongated cells in comparison with the wild type strain and mutant A68 lacked flagella as evidenced by the TEM micrographs (**Figure 1B**). The flagella-less A68 mutant remained motile expressing similar motility as the wild type strain in repeatable experiments. Therefore, observed decreased virulence of this mutant is not connected with motility.

### Ability of *D. solani* Tn5 Mutants to Cause Symptoms on Potato Tubers and Chicory Leaves

**FIGURE 2:**
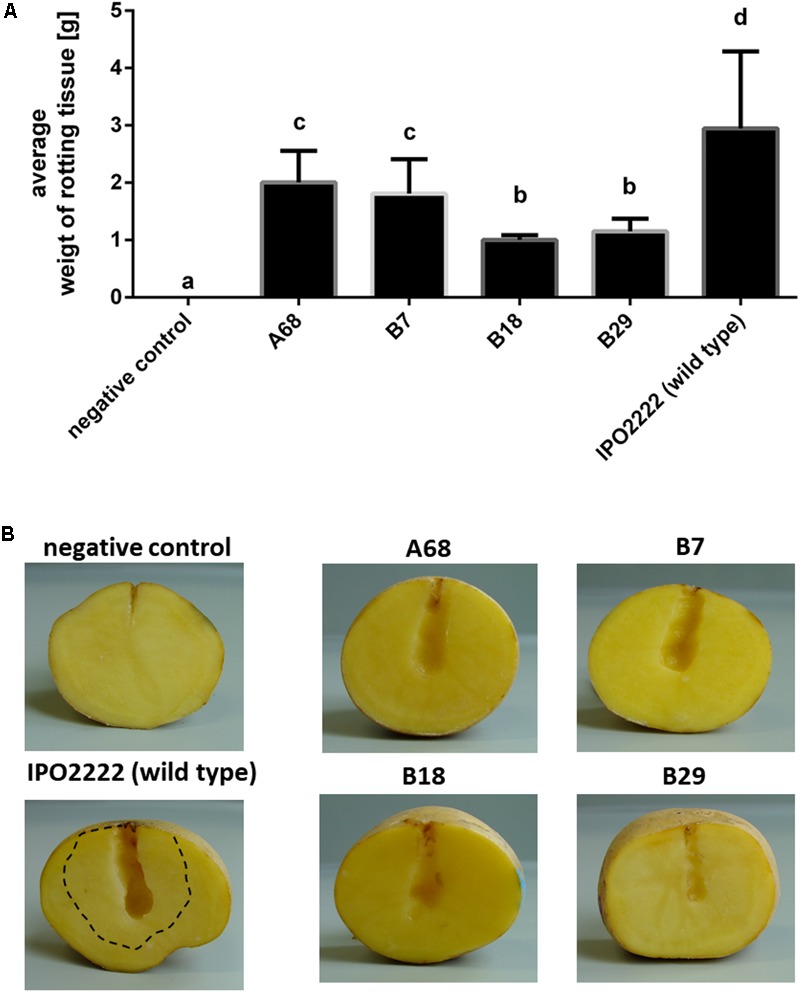
Ability of four *Dickeya solani* IPO2222 Tn5 mutants to cause maceration (rotting) of potato tuber tissue. Quantitative determination of average weight of rotting tuber tissue (in grams) collected after 72 h incubation at 28°C in a humid box. Per mutant, ten individual potato tubers were inoculated in two independent experiments (*n* = 20). Results were considered to be significant at *p* = 0.05 and pair-wise differences were obtained using the *t*-test. Error bars represent standard deviation (SD) **(A)** and visual estimation of symptom expression in potato tubers inoculated with individual IPO2222 Tn5 mutants or wild type (IPO2222, control) in Ringer’s buffer (Merck) after 72 h incubation in a humid box **(B)**. Dotted line in **(B)** shows extent of tissue maceration – softening of plant tissue by the wild type IPO2222 strain.

**FIGURE 3:**
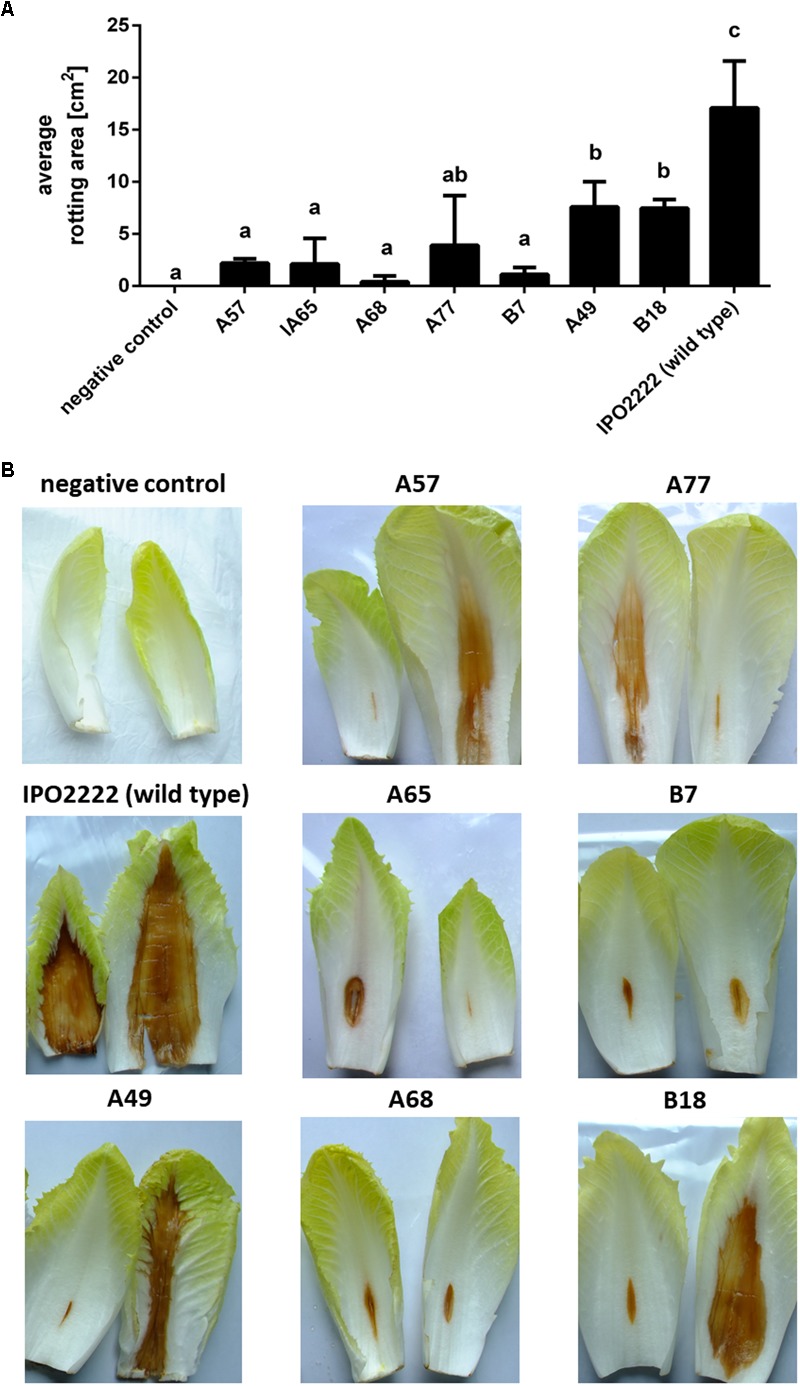
Ability of seven *Dickeya solani* IPO22222 Tn5 mutants to cause maceration of chicory leaves. Quantitative determination of the average area of rotting (in cm^2^) after 48 h incubation at 28°C in a humid box. Per mutant, five individual chicory leaves were inoculated in two independent experiments (*n* = 10). Results were considered to be significant at *p* = 0.05 and pair-wise differences were obtained using the *t*-test. Error bars represent standard deviation (SD) **(A)** and visual estimation of symptom expression in chicory leaves inoculated with individual IPO2222 Tn5 mutants or wild type (IPO2222, control) in Ringer’s buffer (Merck) after 48 h incubation in a humid box **(B)**.

The selected 37 mutants were tested in potato tuber and in chicory leaf assays for altered ability to macerate plant tissue. Four *D. solani* Tn5 mutants (A68, B7, B18, and B29) expressed statistically significant reduction of potato tuber maceration ability (**Figures 2A,B**) and 7 mutants (*viz*. A49, A57, A65, A68, A77, B7, and B18) expressed significantly reduced ability to cause symptoms on chicory leaves in comparison with the wild type strain (**Figures 3A,B**).

### Characterization of *D. solani* Mutants Exhibiting Altered Virulence

A single insertion of the Tn5 transposon was observed in the genome of each of the 8 *D. solani* mutants having an altered plant virulence as assessed by Southern blot analyses and sequencing of the bacterial genomes (data not shown), indicating that the differential GUS activity observed in these mutants was linked to the locus into which the transposon inserted. DNA sequences flanking the inserted Tn5 transposon were determined by sequencing of the genome of each mutant, and were annotated using BLAST at both the DNA and protein level. These loci encoded proteins involved in fundamental bacterial metabolism, secretion of bacteriocins, and chemotaxis as well as hypothetical proteins and proteins with unknown function (**Table 2**).

Most of the mutants with altered plant virulence did not differ from the wild type strain in their ability to catabolize or tolerate various compounds. Six of the mutants (A49, A57, A65, A77, B7, and B29) exhibited the same pattern of catabolize utilization as the wild type strain, whereas mutant A68 gained susceptibility to lithium chloride and mutant B18 lost the ability to utilize D-glucuronic acid (data not shown). Mutants A65, A68, B7, and B18 were additionally tested for production of secreted enzymes using API-ZYM assays. As expected, all Tn5 mutants gained glucuronidase (GUS) enzymatic activity due to the expression of the GUS reporter gene (GUS) located on the reporter transposon but did not otherwise differ from that of the WT strain (data not shown).

### Expression of Oxygen-Responsive Genes of *D. solani in Planta*

The expression of the oxygen-responsive genes identified in mutants with altered plant virulence were assessed in cells inoculated into various potato tissues. Except for mutant A49 which did not express GUS activity when inoculated into tissues of *S. tuberosum*, the expression of this reporter gene in all other tested mutants was induced when cells were inoculated into plant roots, stems, potato tubers and leaves (**Table 3**). Interestingly, mutants A65 and B7 exhibited GUS activity when inoculated into all of the several potato tissues tested, whereas mutants A57 expressed reporter gene activity only when inoculated into leaves and mutants A77 and B18 expressed GUS activity when inoculated into leaves and potato minitubers and mutant B29 exhibited GUS activity only when inoculated into potato stems (**Table 3**).

**Table 3:** Genetic loci of *D. solani* strain IPO2222 Tn5 mutants regulated by the presence of plant tissue of *Solanum tuberosum*.

			Fold induction in contact with *S. tuberosum*			
No	Mutant	Tn5 locus, accession number, protein, predicted function	leaf tissue	stem tissue	root tissue	tuber tissue
1	A49	WP_022634006; LacI/PurR family transcriptional regulator	0	0	0	0
2	A57	WP_022634533; lytic transglycosylase, murein transglycosylase D	15.7	0	0	0
3	A65	WP_022634250; ferric vibriobactin enterobactin ABC transport system ATP-binding protein ViuC/FecE	13.6	1.9	3.6	5.7
4	A68	WP_022633687; hypothetical protein, inner membrane protein	5.0	0	2.4	5.4
5	A77	WP_012883918; colicin transporter, TolR protein	6.45	0	0	1.65
6	B7	WP_023637659; proline-specific permease ProY	7.7	8.0	16.8	5.4
7	B18	WP_022634983; hypothetical protein	2.4	0	0	1.3
8	B29	WP_022634128; hypothetical protein	0	3.6	0	0

**FIGURE 4:**
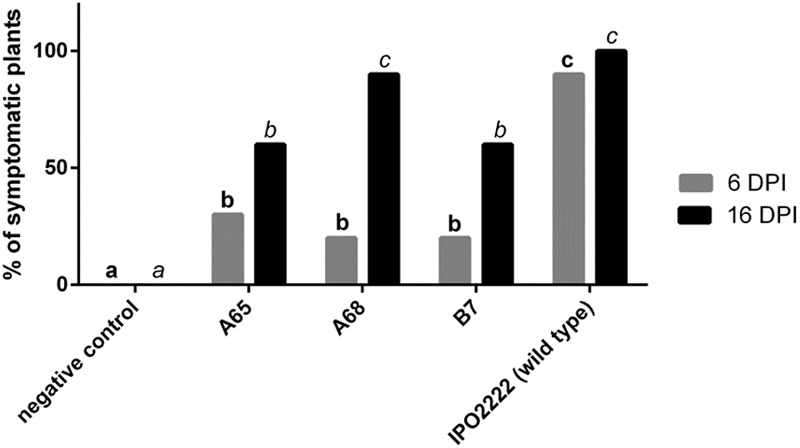
Ability of the three *Dickeya solani* IPO2222 Tn5 mutants to colonize *in vitro* grown potato plants cv. Kondor measured as a percentage of symptomatic plants 6 and 16 days post inoculation (DPI). Two week old plants were inoculated with 10 μl of 10^8^ cfu mL^-1^ (final:10^6^ cfu per plant) suspension of individual *D. solani* Tn5 mutant (treatment) or wild type *D. solani* IPO2222 (control) by applying bacterial suspension on the interspace between the stem base and MS medium of each individual plant. As a negative control 10 μl of sterile 1/4 Ringer’s buffer was used. Each treatment consisted of 10 potato plants grown in individual culture tubes and the entire experiment was repeated independently one time with the same setup (*n* = 20 per treatment). Inoculated plants were visually inspected after 6 and 16 dpi for wilting, typical blackleg symptoms, stem desiccation and/or plant death.

The 8 *D. solani* mutants with reduced ability to cause maceration of potato tubers or chicory leaves differed in their ability to colonize and cause wilting, blackleg symptoms, stem desiccation, and/or plant death as evidence of virulence when inoculated into small *in vitro* grown potato seedlings. Three mutants (A65, A68, and B7) exhibited significantly reduced colonization ability compared to the wild type strain at various times after inoculation. Differences in the apparent virulence of these strains and that of the wild type strain tended to decrease with increasing incubation time; whereas a significantly smaller fraction of plants inoculated with these mutant strains exhibited symptoms compared to the wild type strain after 6 days of incubation, the incidence of symptomatic plants inoculated with the mutants and the wild type strain were more similar when measured after 16 days (**Figure 4**).

## Discussion

Although oxygen availability is one of the key parameters affecting growth of microorganisms in natural environments, the effect of limited oxygen concentration (hypoxia) on the growth, fitness and virulence of *D. solani*, an organism that would frequently encounter such a condition, has not been previously investigated. Like most genera in the *Enterobacteriaceae, Dickeya* are facultative anaerobes and thus able to grow under both anaerobic and aerobic conditions, having both aerobic respiration and anaerobic fermentation (Smid et al., 1993). It is widely considered that these traits are central to the pathogenic success of other SRE such as *Pectobacterium* spp. and *Dickeya* spp. where they experience a broad range of oxygen concentrations depending on the host plant tissue they invade (Lund and Wyatt, 1972; De Boer and Kelman, 1978; Morris et al., 2009). In this study we used a random Tn5- based reporter transposons to identify those genes whose transcription were responsive to oxygen concentrations and test the hypothesis that such oxygen-responsive genes would commonly be required for virulence in potato tubers and aboveground plant tissues that would be expected to vary in oxygen availability.

While the expression of a large percentage of the genes in *D. solani* were interrogated with our reporter transposon, we were surprised that only 1.46% of the mutants exhibited oxygen-dependent transcription. The proportion of oxygen-dependent loci we found in *D. solani* is much lower than the 10% genes of *D. dadantii* strain 3937, a bacterium closely related to *D. solani*, found using micro-arrays to assess transcript abundance (Babujee et al., 2012). Although *D. dadantii* and *D. solani* share many genes, *D. solani* contain several hundred genes absent from the genome of *D. dadantii* (Garlant et al., 2013; Pedron et al., 2014). In addition to the distinct traits these two species may exhibit, they may also this differ in their responsiveness to oxygen variable environments. Methodological differences in the way oxygen-dependent genes were assessed in these two studies may also account for the apparent differences in the frequency of such genes found. Our use of a reporter transposon directly assesses the rate of transcript generation. On the other hand, micro-array analyses assess the abundance of a particular transcript at a given time. It is possible that many oxygen-dependent genes undergo regulation has stepped after transcriptional initiation, such as by influencing transcript stability. Such genes would have the signature of oxygen-dependent transcript abundance but not necessarily that of the rate of transcript initiation, the latter being a measure obtained using the GUS reporter gene system used here. A non-random pattern of insertion of the reporter transposon in our study might also have resulted in a relatively low fraction of insertional events occurring in genes whose transcription was oxygen dependent if those genes were not sites for preferential insertion of the Tn5 vector. We can further hypothesize that due to the fact that the initial screening of the mutants was done on a minimal medium not supporting the growth of putative auxotrophic strains (Winterberg et al., 2005), only prototrophic Tn5 mutants were obtained and analyzed in this study. This may as well explain the differences in the number of oxygen-dependent genes in *D. dadantii* and *D. solani* species reported but more work is needed to tackle this hypothesis.

The apparent proportion of oxygen responsive genes in *D. solani* IPO2222 would indicate that the species would be more capable to cause disease symptoms on host plants under a broader range of conditions. The most successful plant pathogens are expected to be able to cause infection in the plant host under numerous environmental conditions (Alfano and Collmer, 1996; Somssich and Hahlbrock, 1998), and fluctuating oxygen concentrations in different plant species and tissues would certainly be a condition that SRE would be expected to encounter. Therefore, it would be advantageous for plant pathogen to be able to express virulence traits under a wide range of oxygen concentrations. Since *D. solani* is known to be more virulent and widespread than other *Dickeya* spp. (Toth et al., 2011; Czajkowski et al., 2012; Degefu et al., 2013; Tsror et al., 2013) including *D. dadantii* we speculate that it is not merely the fraction of genes that are oxygen regulated in *D. dadantii* that contribute to its success as a pathogen but instead the identity of those genes. Indeed, the majority of the 37 insertional mutants for which relatively large oxygen-dependent rates of transcription were observed did not individually have measurable effects on virulence, and probably represent the myriad of genes involved in simple aerobic versus anaerobic metabolism. It is noteworthy, however, that eight insertional mutants (about 20% of those exhibiting relatively large oxygen dependent transcriptional initiation) did substantially influence the virulence of *D. solani* to one or more plant hosts or conditions (**Table 2**). Surprisingly, none of these 8 mutants had an insertion in genes encoding for well-described virulence factors used by *Dickeya* spp. to infect plant host (Reverchon and Nasser, 2013), revealing that virulence traits in the SRE and particularly *D. solani*, are not fully elucidated. Among these novel virulence genes are those encoding a LacI/PurR family transcriptional regulator (A49), lytic transglycosylase (A57), ferric vibriobactin enterobacterin ABC transport ATP-binding protein ViuC/FecE (A65), colicin transporter (A77), proline-specific permease ProY (B7) and three others coding for hypothetical proteins (A68, B18, and B29).

Some support for payroll of LacI family transcriptional regulators as virulence factors has previously been obtained. In *D. dadantii* strain 3937, a close-relative to *D. solani*, 80 LacI family transcriptional regulators were characterized from which several were involved in infection process of host plants (Van Gijsegem et al., 2008). For example, *lfaR* and *lfcR* negative mutants expressed reduced virulence on chicory, *Saintpaulia* sp. and *Arabidopsis*. The LacI family regulator disrupted by the Tn5 in the A49 mutant possess 96% identity with the LacI/PurR transcriptional regulator of *D. dadantii* 3937 described by Van Gijsegem et al. (2008) which indicates that in both 3937 and IPO2222, this regulator plays a role in infection process. LacI/PurR homologs were found also in *D. zeae, D. dianthicola*, and *D. chrysanthemi* but little is known about its function in these *Dickeya* species.

Likewise, several regulators of the LacI family have already been shown to be involved in bacterial virulence against animals. For instance, in *Pseudomonas aeruginosa* strain PAO1, PtxS – LacI family member is a negative regulator involved in activation cascade of exotoxin A production (Swanson et al., 2000) and in *Streptococcus pneumoniae*, two LacI family regulators (RegM and RegR) are involved in virulence by modulating adhesion of bacterial cells to their hosts (Chapuy-Regaud et al., 2003).

Lytic transglycosylases are bacterial enzymes that act on peptidoglycan and possess the same substrate specificity as lysozyme (Scheurwater et al., 2008). They have been associated with the type III and type IV secretion systems (T3SS and T4SS) of Gram-negative phytopathogens and their proposed mode of action involve enlargement of pores in peptidoglycan where the secretion machinery needs to be accommodated and localized, which contributes to the effective secretion of virulence factor from bacterial to host cell (Koraimann, 2003). In plant pathogenic bacteria such as *Ralstonia solanacearum, Xanthomonas* sp. *Erwinia amylovora* and *Pseudomonas syringae* several lytic transglycosylases have been described and linked with the T3SS (Oh et al., 2007). For example, in *E. amylovora* a lytic transglycosylase gene was found to be up-regulated *in planta* and to contribute to virulence (Zhao et al., 2005). In *R. solanacearum* genes coding for lytic transglycosylases are up-regulated by the same activator that activate genes encoding T3SS proteins (Occhialini et al., 2005). Similarly, in *Caulobacter crescentus* lytic transglycosylase PleA is required for assembly of pili and flagellum at the cell pole (Viollier and Shapiro, 2003). While prior to this study no data linked lytic transglycosylases to the virulence of *Dickeya* spp., but our results suggest that these enzymes may play a central role in virulence and colonization of potato plants in *D. solani* given the large virulence defects in an mutants in this study.

The *viuC*/*fecE* gene product is a member of the FecBCDE citrate-dependent iron (III) transport system in *D. solani*. Due to its minimal solubility in soils, iron is one of the most limited nutrients available in nature. Iron ions are required for virulence of most animal and plant pathogenic bacteria (Expert and O’Brian, 2012) including *D. dadantii* strain 3937, in which iron acquisition serves as a virulence factor (Enard et al., 1988). Low local iron concentrations inside plants inhibits infections by phytopathogenic bacteria (Pandey et al., 2016). It is thus noteworthy that mutant A65, defective in a FecBCDE citrate-dependent iron (III) transport system, while able to cause some soft rot on potato exhibited greatly reduced rotting symptoms on chicory leaves in comparison with the wild type strain and reduced colonization ability on potato seedlings.

The colicin-like bacteriocins are well-characterized antibacterial proteins that are secreted and active against closely related bacteria belonging to the same species (Chavan and Riley, 2007). These proteins are widely distributed in Gram-negative bacteria including those causing disease in plants (e.g., *Agrobacterium* spp., *Pectobacterium* spp., and *Xanthomonas* spp.) (Grinter et al., 2012). The bacteriocins are believed to contribute to the competitiveness of pathogens in environmental niches that would typically contain a mixture of other antagonistic bacterial species (Riley and Gordon, 1999). Several bacteriocins were reported to be produced by soft rot *Pectobacterium* spp. but only limited knowledge exists on bacteriocin production by *Dickeya* spp. including *D. solani*. Garlant (2015) described gene clusters encoding bacteriocins in the genomes of *D. solani* strains Ds0432-1 and IPO2222. In plate assays performed *in vitro*, these bacteriocins were active against closely related *P. carotovorum* subsp. *carotovorum* strain SCC1 and *P. atrosepticum* strain SCRI 1043 but not against other *Dickeya* spp. (Garlant, 2015). Likewise, we previously have shown that bacteriocin production has no impact on the competition between *D. solani* and *D. dianthicola* biovar 1 and 7 strains during infection of potato plants and tubers (Czajkowski et al., 2012) but may play a role in environments in which other pectinolytic species are present. The decreased virulence of mutant A77 on chicory leaves, a habitat in which mixed bacterial communities would be expected, supports a model that bacteriocins are important fitness factors in a setting where competitive bacteria are present.

Proline plays various roles in bacterial cellular processes including osmoregulation, protein stability, stress resistance, as well as biosynthesis of secondary metabolites having antibacterial properties (Christgen and Becker, 2017). In *D. dadantii* strain 3937 proline acts as osmoprotectant enabling the recovery of bacterial growth after exposure to high osmolarity during infection (Gouesbet et al., 1995). Likewise, in *Salmonella enterica* serovar Typhimurium a proline-specific permease allows utilization of proline as a sole source of both carbon and nitrogen (Liao et al., 1997). As the B7 mutant of *D. solani* exhibited reduced colonization ability on potato seedlings and was impaired in maceration of potato tuber and chicory leaf tissues it is clear that proline metabolism is essential for full virulence.

Even though none of the described-above mutants have the Tn5 insertion in the locus coding for known pathogenicity determinants of *Dickeya* spp., these loci apparently increase its ecological fitness, in complex and diverse environments that experience variations in oxygen content. A previous study that identified thermo-regulated genes in *D. solani* strain IFB0099 that influence the virulence of this strain (Czajkowski et al., 2017a) also found many to be involved in primary bacterial metabolism and not for previously identified virulence factors such as secreted pectinolytic enzymes, cellulases and proteases. Thus the findings of a contribution of both oxygen and temperature regulated genes to virulence in *Dickeya* spp supports the conjecture of Salmond (1994) that regulation of virulence in soft rot *Enterobacteriaceae* is a complex network of interactions involving fundamental bacterial metabolism as well as specific regulation of production and secretion of factors engaged strictly in the virulence program (Salmond, 1994).

In conclusion, this study is the first step on the way to a better understanding of how oxygen level affects expression of virulence and fitness-related genes, which might also influence the disease severity under different environmental conditions. To fully explore the oxygen-dependent gene expression regulation in *D. solani* additional studies are required and are now being done. The work with deletion (knockout) mutants in the candidate genes of *D. solani* is in progress to find the molecular basis of oxygen-dependent gene expression regulation of virulence- and ecological fitness-related genes.

## Author Contributions

RC: conceptualization, formal analysis, funding acquisition, and writing, reviewing, and editing the manuscript. WL and RC: data curation, project administration, and writing the original manuscript. WL, JF-K, SJ, MN, PC, and RC: investigation and methodology. PC and RC: resources. RC and MN: software and supervision. WL, JF-K, SJ, and MN: validation. WL, JF-K, SJ, MN, and PC: visualization.

## Conflict of Interest Statement

The authors declare that the research was conducted in the absence of any commercial or financial relationships that could be construed as a potential conflict of interest.

## Footnotes

1 https://genesilico.pl/meta2/acl_users

2 http://www.ncbi.nlm.nih.gov
