# Supplementary material for: Oxygen Availability Influences Expression of Dickeya solani Genes Associated With Virulence in Potato (Solanum tuberosum L.) and Chicory (Cichorium intybus L.)
Source: Front Plant Sci. 2018 Mar 21;9:374. doi: 10.3389/fpls.2018.00374 (PMC5872005; doi:10.3389/fpls.2018.00374)

Supplementary Material

Oxygen availability influences expression of *Dickeya solani* genes associated with virulence in potato (*Solanum tuberosum* L.) and chicory (*Cichorium intybus* L.)

Wioletta Lisicka [1], Jakub Fikowicz-Krosko [1], Sylwia Jafra [1], Magdalena Narajczyk [2], Paulina Czaplewska [3] and Robert Czajkowski [1, *]

[1] University of Gdansk, Intercollegiate Faculty of Biotechnology of University of Gdansk and Medical University of Gdansk, Department of Biotechnology, Gdansk, Poland

[2] University of Gdansk, Faculty of Biology, Laboratory of Electron Microscopy, Gdansk, Poland

[3] University of Gdansk, Intercollegiate Faculty of Biotechnology of University of Gdansk and Medical University of Gdansk, Core Facility Laboratories, Laboratory of Mass Spectrometry, Gdansk, Poland

*** Correspondence:**
Dr. Robert Czajkowski
robert.czajkowski@biotech.ug.edu.pl

Supplementary Figure 1. Growth rate of a parental (IPO2222) and eight Dickeya solani IPO2222 Tn5 mutant strains with altered phenotypes. The growth rate of Tn5 mutants was determined in M9 medium supplemented with 0.4% glucose at 28 °C by measuring the optical density (OD600) every hour for a period of 16 h. Bacterial densities were estimated from the OD reads, assuming that OD600 is equal to 10^8 cfu mL^{-1}. Six replicates of each strain were analyzed per time point and the results were averaged. M9 medium supplemented with 0.4% glucose not inoculated with bacteria was used a negative control.

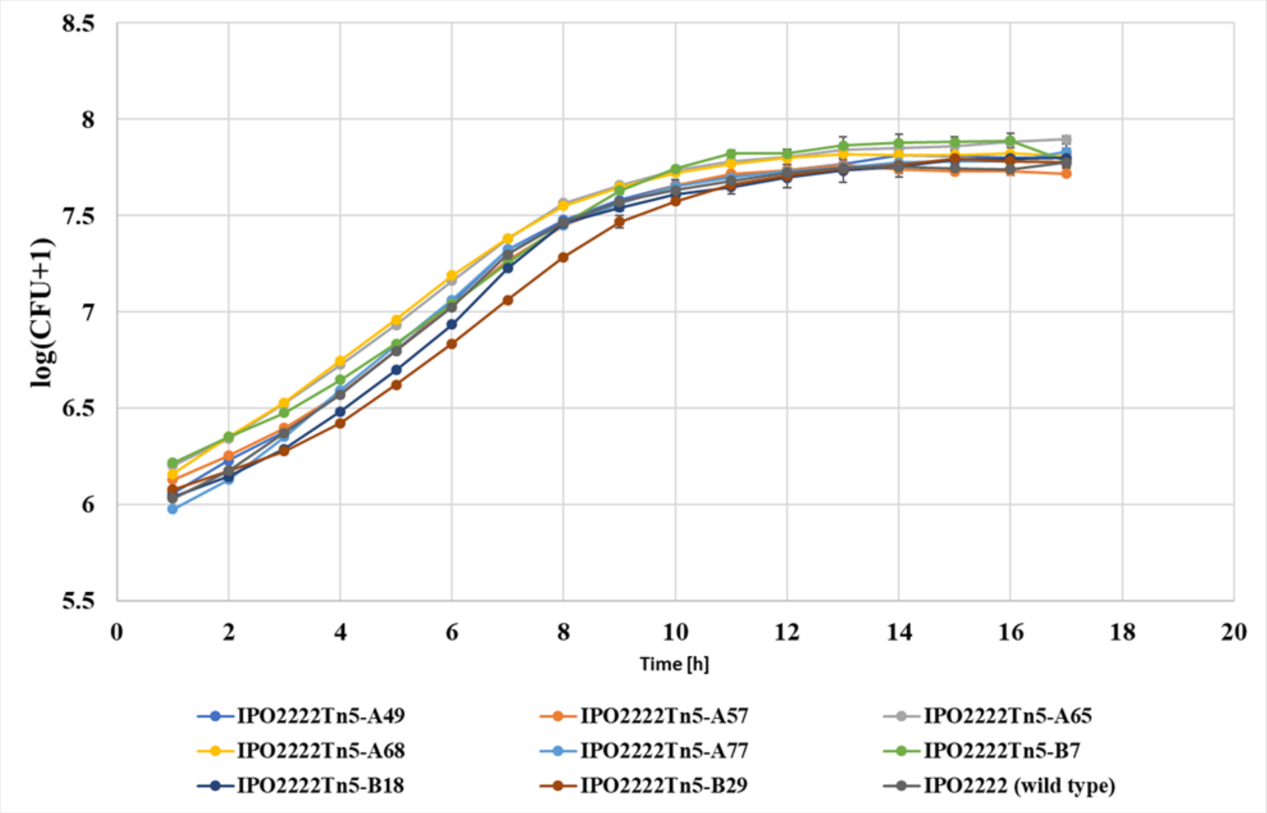

Supplement: Supplementary file 1 [file Image_1.PDF]
