# Supplementary material for: Oxygen Availability Influences Expression of Dickeya solani Genes Associated With Virulence in Potato (Solanum tuberosum L.) and Chicory (Cichorium intybus L.)
Source: Front Plant Sci. 2018 Mar 21;9:374. doi: 10.3389/fpls.2018.00374 (PMC5872005; doi:10.3389/fpls.2018.00374)

Supplementary Material

Oxygen availability influences expression of *Dickeya solani* genes associated with virulence in potato (*Solanum tuberosum* L.) and chicory (*Cichorium intybus* L.)

Wioletta Lisicka [1], Jakub Fikowicz-Krosko [1], Sylwia Jafra [1], Magdalena Narajczyk [2], Paulina Czaplewska [3] and Robert Czajkowski [1,*]

[1] University of Gdansk, Intercollegiate Faculty of Biotechnology of University of Gdansk and Medical University of Gdansk, Department of Biotechnology, Gdansk, Poland

[2] University of Gdansk, Faculty of Biology, Laboratory of Electron Microscopy, Gdansk, Poland

[3] University of Gdansk, Intercollegiate Faculty of Biotechnology of University of Gdansk and Medical University of Gdansk, Core Facility Laboratories, Laboratory of Mass Spectrometry, Gdansk, Poland

*** Correspondence:**
Dr. Robert Czajkowski
robert.czajkowski@biotech.ug.edu.pl

Supplementary Figure 2. Whole-cell MALDI-TOF protein mass fingerprints of *D. solani* IPO2222 wild type and 8 selected Tn5 mutants in ferulic acid matrix. Protein mass fingerprint patterns obtained in positive ion mode in the mass range 4000 - 20 000 Da.

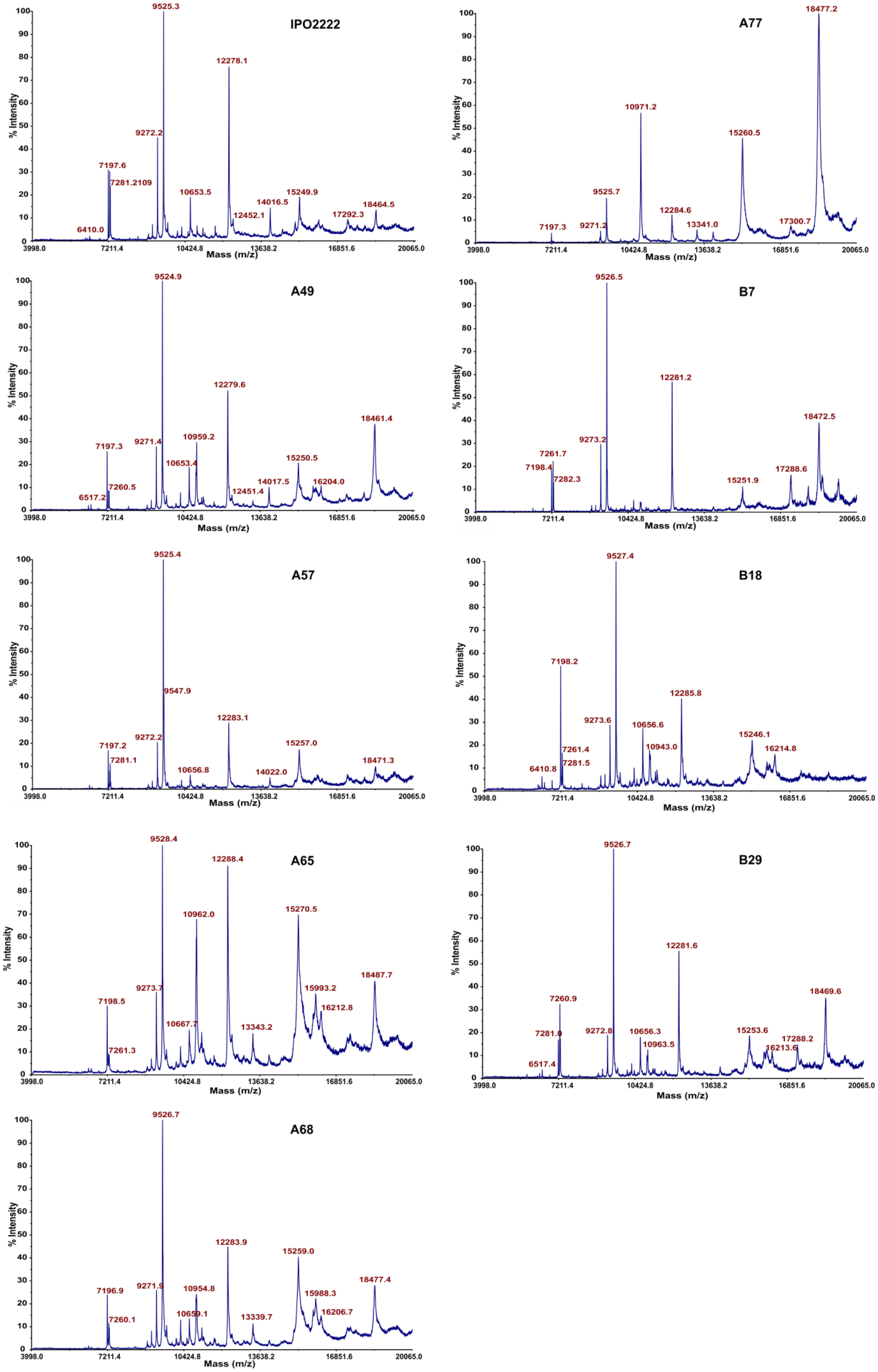

Supplement: Supplementary file 2 [file Image_2.PDF]
